# Tuning Surface Morphology of Fluorescent Hydrogels Using a Vortex Fluidic Device

**DOI:** 10.3390/molecules25153445

**Published:** 2020-07-29

**Authors:** Javad Tavakoli, Colin L. Raston, Youhong Tang

**Affiliations:** 1 Centre for Health Technologies, School of Biomedical Engineering, Faculty of Engineering and Information Technology, University of Technology Sydney, Ultimo NSW 2007, Australia; javad.tavakoli@uts.edu.au; 2 Institute for NanoScale Science and Technology, College of Science and Engineering, Flinders University, Bedford Park, SA 5042, Australia; colin.raston@flinders.edu.au

**Keywords:** vortex fluidic device, fluorescent hydrogels, aggregation-induced emission, self-adhesion, fluorescent property, surface morphology

## Abstract

In recent decades, microfluidic techniques have been extensively used to advance hydrogel design and control the architectural features on the micro- and nanoscale. The major challenges with the microfluidic approach are clogging and limited architectural features: notably, the creation of the sphere, core-shell, and fibers. Implementation of batch production is almost impossible with the relatively lengthy time of production, which is another disadvantage. This minireview aims to introduce a new microfluidic platform, a vortex fluidic device (VFD), for one-step fabrication of hydrogels with different architectural features and properties. The application of a VFD in the fabrication of physically crosslinked hydrogels with different surface morphologies, the creation of fluorescent hydrogels with excellent photostability and fluorescence properties, and tuning of the structure–property relationship in hydrogels are discussed. We conceive, on the basis of this minireview, that future studies will provide new opportunities to develop hydrogel nanocomposites with superior properties for different biomedical and engineering applications.

## 1. Introduction

Hydrogels, appealing soft biomaterials with viscoelastic characteristics, are crosslinked polymeric networks that maintain distinct 3D structures while absorbing water. The capability of hydrogels to create self-assembled structures, along with their high swelling properties, makes them attractive for different applications [1,2,3]. Over decades, different crosslinking methods have been identified for the fabrication of chemically stable or degradable hydrogels for various applications [4,5,6]. Of particular interest, smart hydrogels have been extensively utilized for the sustained release of pharmaceutics with controlled pH [7,8,9], electric fields [10], osmosis [11], molecular structure [12,13], and temperature [14,15,16,17], regulating their swelling properties. In the meantime, numerous studies have been performed to improve the physical, mechanical, and biomedical properties of hydrogels, making them attractive for a wide range of applications [18,19].

In the past few decades, technologies have been acquired with modifications from different engineering fields to advance hydrogel design and control the architectural features at the micro- and nanoscale [20,21,22]. The most frequently used and simple technique for producing hydrogel films is molding [23]. Simple molding leads to the construction of hydrogels with an almost smooth surface, lacking surface architectural features; however, molding materials may enable control of the surface energy of the final hydrogels [24,25]. The creation of micropatterned hydrogels is also achieved by soft lithography using elastomers [26]. Although this approach has been used for the fabrication of both physically and chemically crosslinkable hydrogels, detachment of hydrogels from the mold is a major drawback, affecting the quality of the patterns [27].

Photopatterning, a popular method for the microfabrication of electromechanical systems, involves light irradiation through a patterned mask, crosslinking certain hydrogel areas to ultimately form the desired micropatterns [28]. This technique applies to photo-crosslinkable hydrogels with no control over the depth of crosslinking. Recent laser-based technologies, including stereolithography, digital light projection, and two-photon polymerization, are alternative photopatterning systems advancing the fabrication of micropatterned hydrogels with a higher resolution, where no physical mask is used, contrasting with photomask patterning counterparts [29]. Lithographic patterning techniques relying on the selective elimination of materials to create topographies with preferred shape and size do not need further assembly steps. Well-established methodologies incorporating precise engineering control have made lithographic patterning techniques capable of developing 3D entities at the nanoscale [30,31,32,33,34]. Using the techniques, a variety of 3D constructs have been developed with different applications in biomedical devices [35,36,37,38], optoelectronics and electronics [39,40,41,42], microelectromechanical systems [43,44], and sensors [45,46,47,48]. However, these methods combine high costs with a relatively slow process, making the duration of production problematic.

Microfluidic techniques have also been used for the fabrication of hydrogels with limited simple shapes, i.e., sphere, core-shell, fibers, and more complex morphology when combined with photolithography and micromachining techniques [49,50]. Moreover, control of hydrogel composition can be achieved using multichannel microfluidic technologies with different flow rates and chemicals [51,52]. Upon the introduction of microfluidic technologies, the preparation of hydrogel-based platforms resembling the intrinsic complexity of different tissues was advanced dramatically and the biological relevance of cell models was improved [53,54,55,56]. Recently, researchers are taking advantage of the use of microfluidic platforms to develop hydrogel-based cell culture media to precisely perform cell assays [57,58]. These platforms, in combination with high-resolution and real-time imaging techniques, have resulted in the development of nanodevices suitable for studying the impact of morphology on cell behavior and different biomedical applications with accurate and stable control of the culture environment [59,60,61]. The major challenges with the microfluidic approach are clogging, limitation of possible shapes, and the infeasible implementation of a reasonable batch production time. Apart from the specific limitations relevant to the currently available methods to create micropatterned hydrogels, lack of control over the mixing process at the molecular level is a severe drawback, limiting possible applications. Current methods involve distinct chemical mixing and micropatterning routes before hydrogel construction, i.e., simple mixing using a magnetic stirrer, limiting control over the final properties of micropatterned hydrogels.

In this minireview, we aim to introduce a novel, cost-effective, and easy-to-use technique for the fabrication of micropatterned hydrogels using a vortex fluidic device (VFD). The VFD allows sufficient micromixing and transformation of chemicals under shear stress with concurrent manipulation of the hierarchical structure. The VFD enables control of the surface micropattern and tunes the properties of hydrogels, simultaneously ultimately creating micropatterned hydrogels with superior physicochemical and mechanical properties. The application of a VFD to develop hydrogel constructs with tunable bulk properties and different surface morphologies is a relatively new approach and further studies may enhance our knowledge about the mechanism of hydrogel formation, thus, expanding the applications.

## 2. Hydrogel Constructs under Thin Film Formation

### 2.1. Vortex Fluidic Device (VFD)

**Figure 1:**
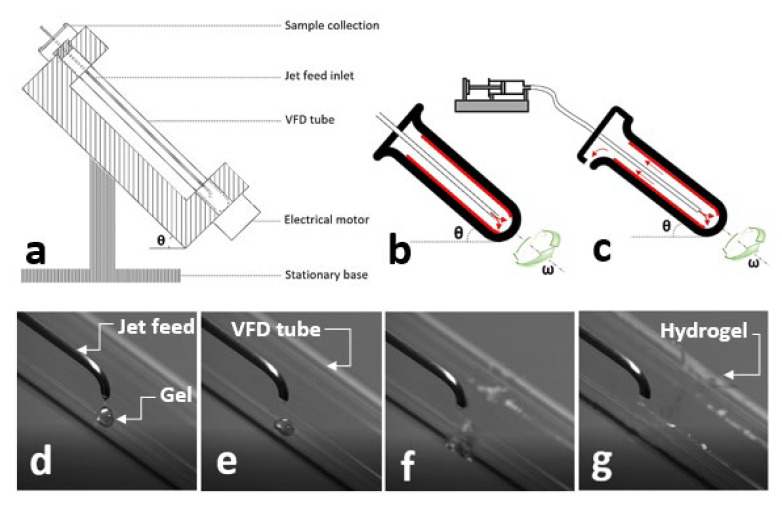
(**a**) Schematic drawing of a vortex fluidic device (VFD) indicating its compartments. Schematic drawings represent (**b**) confined and (**c**) continuous flow modes of the VFD operation. (**d**–**g**) High-speed camera images captured during the injection of gel solution into a rotating VFD tube in a confined mode at different time points. θ is the title angle (^o^) and ω is the rotation speed (rpm).

The VFD is a microfluidic platform consisting of a glass tube rotating rapidly to provide a dynamic thin film for chemical reactions (Figure 1a) [62]. The thin layer is generated over the rotating surface, with the rotation precisely controlling the thickness of the layer and imparting shear stress for micromixing. The tilt angle of the tube is adjustable from 0° to almost 90°, within which the tilt angle of 45° has been shown to contribute effectively to the optimization of chemical transformation. It is possible to conduct an experiment using a VFD with two different modes of operation. Filling a closed-end VFD tube with a finite amount of chemicals to collect the final product at the end of the reaction is known as a confined mode of operation (Figure 1b). For a continuous flow mode of operation, chemicals are constantly injected into a rotating VFD tube, with the final product collecting at the top of the tube (Figure 1c).

The VFD is a relatively low-cost research platform assisting efficient micromixing of chemicals and reactions, material synthesis, and both top-down and bottom-up strategies for the manufacture of nanoscale materials with complex structures, suggesting a range of benefits over conventional methods [63,64,65]. We successfully utilized a VFD for one-step fabrication of several physically and ionically crosslinked hydrogels with simultaneous control over their properties and surface morphology [66]. Of particular significance, we prepared fluorescent probes with excellent stability and high fluorescent intensity compared to their traditionally prepared counterparts, to ultimately fabricate fluorescent hydrogels with enhanced properties [67]. Fabrication of hydrogels using a VFD involves the injection of a gel solution into the crosslinking agent via the jet feed inlet (Figure 1d–g), with the rotation speed of the VFD tube precisely controlling the properties and morphology.

With a view to exploring the formation of micropatterned hydrogels using a VFD, different case studies were considered. The results reported in this review include physically crosslinkable hydrogels only, while chemically crosslinkable hydrogels are still under investigation. We hypothesized that the VFD is proficient in the one-step fabrication of hydrogels with different architectural features and surface morphologies. To investigate this concept, polyvinyl alcohol (PVA) was selected, representing hydrogels with hydrogen bonds contributing to their network formation.

### 2.2. Bulk Hydrogels

Performing sol–gel processing with a significant reduction in processing time and consumption of reagents can ultimately permit the preparation of hydrogels in bulk. One study reported the preparation of silica hydrogel using a VFD without the need for auxiliary reagents (solvent, acid, or base), promoting a new route for green chemistry. It was reported that the condensation of VFD-driven silica gel was greater (95%) than that achieved with traditional batch processing (89%). A significant reduction in gelation time (<30 min) was another advantage associated with the use of the VFD [68]. The mechanism of formation of the urea-based gels and the effect of shear stress generated in a VFD tube on their local and bulk structure was studied using a combined VFD and a neutron scattering system. This typical study represents an original platform for future studies designed to understand the impact of different external stimuli on the mechanism of formation and properties of self-assembled gel networks [69].

### 2.3. Surface Morphology

**Figure 2:**
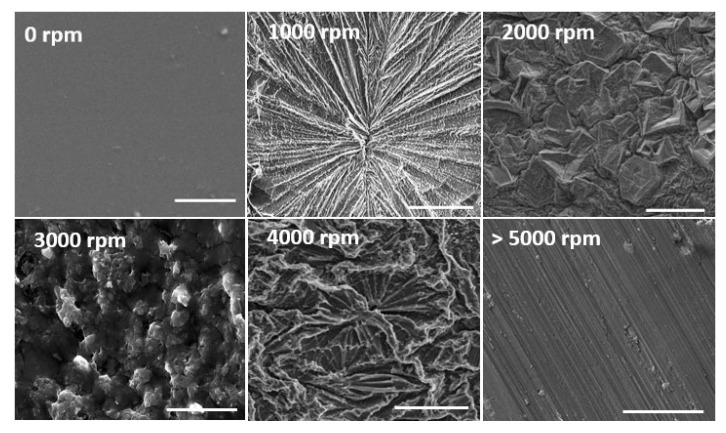
Surface morphologies of VFD-driven polyvinyl alcohol (PVA) hydrogels formed at different rotation speeds of the VFD tube. Scale bars are 50 μm. Images reproduced with permission from [66], copyright (2020) Springer.

Our investigations involving the fabrication of PVA hydrogels using a VFD revealed interesting results. We found that the injection of a PVA solution (5–10% *w/w*) into a rotating VFD tube containing borax solution (100 mM), using a 2 mm jet inlet, resulted in the creation of PVA hydrogel films, with their surface morphologies strongly dependent on the rotation speed of the VFD tube. Borax solution has been used in different studies as a crosslinking agent to induce hydrogen bonds, creating hydrogels for various biomedical applications [70,71]. As observed, depending on the rotation speed, PVA hydrogel films were fabricated with different surface morphologies including radial (1000 RPM) and parallel (5000 RPM) grooves, flowerlike patterns (4000 RPM), and filling with irregular (2000 RPM) and spherical (3000 RPM) shaped particles (Figure 2). The mechanism of the formation of PVA hydrogels with different surface morphologies has been fully discussed previously [31]. Briefly, variation of the magnitude of the centrifugal forces generated at different rotational speeds was likely responsible for the manipulation of different surface textures [66].

**Figure 3:**
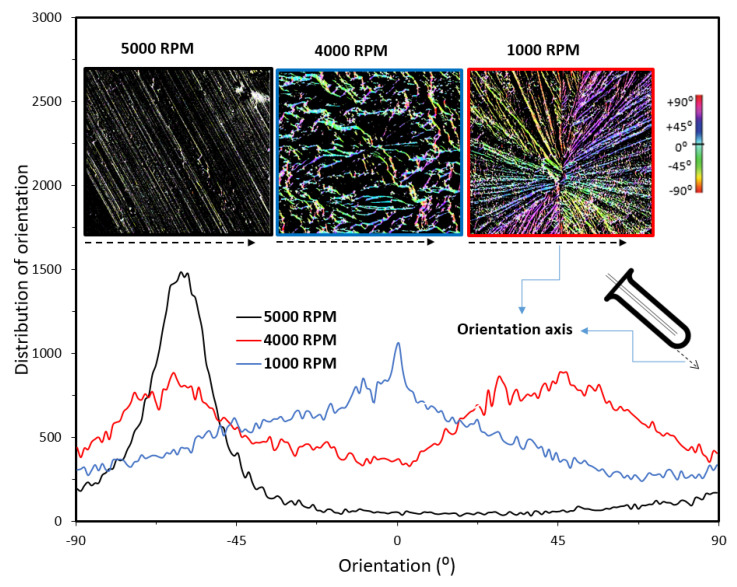
Distribution of the orientation of surface patterns in VFD-driven PVA hydrogels with different preferential directionalities. Insets: a VFD tube and color-survey images indicating the orientations of fibers relative to the orientation axis, denoted by black dotted arrows.

Our further quantitative analysis, using FIJI software, identified specific directionalities with arranged patterns for PVA hydrogels that were fabricated using a VFD (Figure 3). Although two major directions for the pattern orientations at ±45° were observed when the rotation speed was 1000 RPM, only one direction of orientation (−45°) was found for those prepared at 5000 RPM. When the rotation speed of the VFD tube was 4000 RPM, a Gaussian distribution for patterns with their maximum distribution occurring at 0° was observed. The orientations were measured relative to the wall of the VFD tube (Figure 3—denoted by dotted black arrows). Compared to the traditionally prepared PVA with no preferential surface directionality, the surface patterns of the VFD-driven PVA hydrogels were not randomly organized. Overall, VFD is a fast technique with simultaneous mixing and crosslinking in a one-step process, facilitating the implementation of different surface patterns at the microscale. A VFD, compared to other technologies to prepare hydrogels with different surface morphologies, is less expensive, making the preparation approach relatively cheap. The use of other technologies for the creation of organized surface patterns requires multistep adjustments that are time-consuming and involve a greater cost. Building on the preliminary results presented in the current minireview, future studies are required to identify the contribution of rotational speeds, tilt angle, jet feed diameter, and the rate of jet feed displacement on the morphological properties of VFD-driven hydrogel nanofibers.

An understanding of the formation mechanism of the VFD-driven PVA hydrogel film was published in our previous study. Briefly, the injection of the PVA solution into the VFD rotating tube led to the formation of a core-shell structure, where the outer regions of the gel were crosslinked earlier than the bulk. The distribution of the centrifugal force, which is proportional to the rotation speeds, resulted in the formation of texture on the surface of the PVA hydrogel film, leading to the formation of unique surface morphology. Our results demonstrate a novel technique, facilitating a one-step fabrication of PVA hydrogels with different surface morphologies. However, more studies are required to examine the effectiveness of the proposed method for the fabrication of a series of hydrogels, with their creation relying on the formation of hydrogen bonds including, but not limited to, PVA, gelatin, starch, cellulose, guar gum, and their composites [27,28,29]. Another direction for future studies is to develop an understanding of the mechanism of the formation of hydrogels.

### 2.4. Fluorescent Hydrogels

Fluorescent hydrogels, compared to those with a conventional gel structure, can emit light. This feature makes them attractive for advanced applications in regenerative medicine, cell imaging, biochemical sensing, and biomaterials [72,73,74,75]. Conventional routes to fabricate fluorescent hydrogels include physical mixing of a gel with fluorescent dyes before the crosslinking and soaking of a crosslinked hydrogel in a dye solution. The introduction of fluorescent dyes affects the chemical and mechanical stability of hydrogels, as the hydrophobic feature of fluorescent dyes can destabilize the hydrogel network with dye leaching. The fabrication of fluorescent hydrogels with high fluorescence intensities also calls for a high concentration of dyes that can lead to aggregation causing quenching (ACQ). ACQ is a major barrier to the creation of bright fluorescent hydrogels, limiting their application in advanced strategies [76]. The current innovation of aggregation-induced emission fluorogens (AIEgens) has resulted in the synthesis of a series of modern fluorescent dyes exhibiting higher emission when aggregated. Their outstanding photostability, high diversity of molecular structures, and high sensitivity are strong advantages in terms of overcoming the major drawbacks [77,78,79]. Recent studies have shown AIEgens enable hydrogel characterization, that is, visualization of crystalline regions, and accurate measurement of the swelling and degradation processes of hydrogels, features that were not previously achievable [80,81,82]. While AIEgens resolve the issue of creating brighter fluorescent hydrogels at the microscale, i.e., fluorescent nanoparticles, their efficacy in enhancing the fluorescence properties of materials at the macroscale is still under investigation [83,84]. To address this issue, several studies have suggested the use of AIE polymers to develop more efficient fluorescent materials [85]. Of particular significance, hyperbranched AIEgens with unique chemical structures that can simultaneously control the fluorescent and mechanical properties of hydrogels have attracted attention. Unfortunately, chemical routes to synthesize hyperbranched AIEgens involve complex and multistep chemistry that makes the processes expensive, time-consuming, and difficult to perform, constraining large-scale production [86,87,88]. Specifically, the control of gelation, size tuning, and the solubility of conjugated polymers with hyperbranched architecture during direct polymerization or postfunctionalization have remained challenging [89].

**Figure 4:**
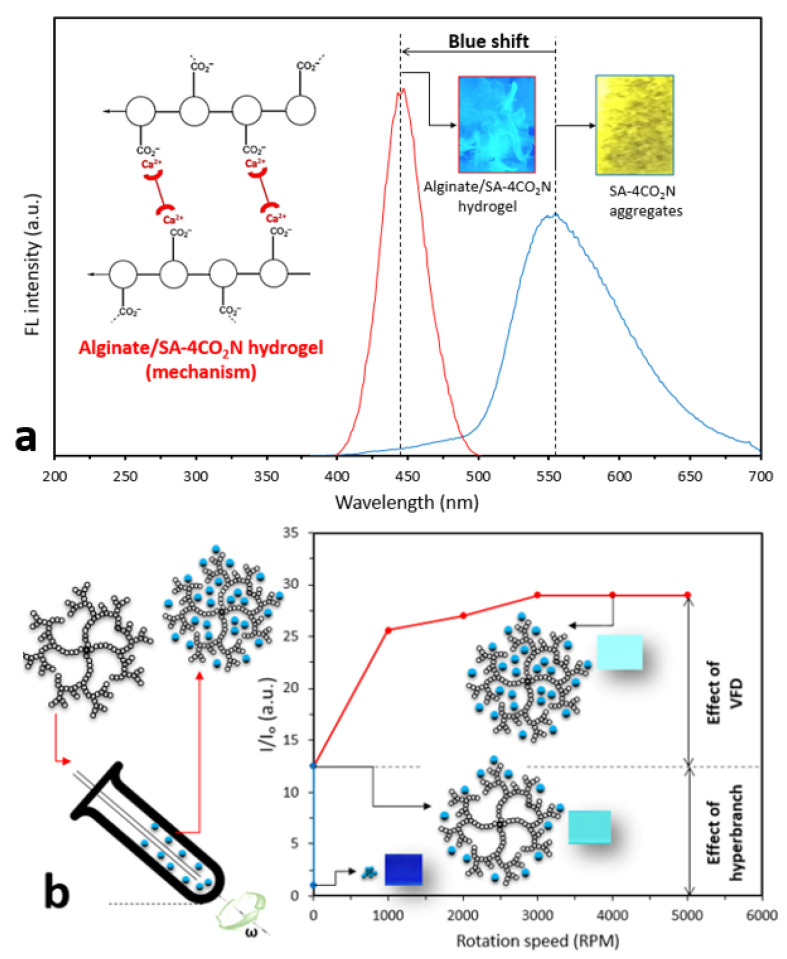
(**a**) Formation of alginate fluorescent hydrogel with calcium ion-sensitive aggregation-induced emission fluorogens (AIEgens) (inset: schematic drawing of formation of the alginate-AIEgen network with fluorescence properties; camera images captured from the fluorescent hydrogel and AIE aggregates in the presence of Ca^2+^ ions excited at 350 nm under a UV light). (**b**) Schematic drawing of the formation of AIEgen-hyperbranched assembly and change in the relative fluorescence intensity of the assembly and AIEgen alone using a VFD. (Inset: schematic drawing of the formation of the AIEgen-hyperbranched assembly, camera images captured from the assembly and AIE aggregates excited at 350 nm under a UV light). (**b**) is reproduced from reference [67] with modifications.

In our previous study, we used SA-4CO_2_Na (20 μM) as an AIEgen to monitor the release of calcium from alginate hydrogels (2% *w/w*) to ultimately characterize swelling and degradation processes. SA-4CO_2_Na aggregates in the presence of Ca ions emitting yellow light at 550 nm. We further prepared a solution containing alginate and SA-4CO_2_Na and fabricated an alginate hydrogel by crosslinking in the CaCl_2_ solution. Subsequently, a fluorescent alginate film was prepared, in which a blue shift was observed due to the disruption of the intramolecular hydrogen bond in SA-4CO_2_Na during the formation of the hydrogel (Figure 4a). Our further investigation revealed that, since the binding constant, as measured by isothermal titration calorimetry between SA-4CO_2_Na and Ca^2+^ (K_d_ = 9.83 × 10^−4^), was almost the same as that of Alginate-SA-4CO_2_Na + Ca^2+^(K_d_ = 8.95 × 10^−4^), SA-4CO_2_Na aggregates during the gelation process within the alginate structure (Figure 4a inset). Therefore, collecting the fluorescent spectra in situ in a VFD tube can inform hydrogel formation, as evidenced by a blue shift for the peak of the fluorescent spectrum.

We recently developed a technique to tune the size of AIEgens using a VFD, leading to the creation of highly fluorescent nanoAIEgens with sizes ranging from 10 to 80 nm [90]. Motivated by this study, we successfully proposed a new methodology to readily prepare AIEgen-hyperbranched assemblies based on diffusion of AIEgens into the hyperbranched structure. TPE-2BA (20 μM), a derivative of tetraphenylethylene with two boronic acid groups, and bis-MPA polyester-64-hydroxyl (0.01, 0.25, 0.5, and 1 mM) were used as AIEgen and hyperbranched polymer. We found that VFD facilitated the creation of an AIEgen-hyperbranched assembly with tunable size and significant enhancement of the fluorescent properties of the assembly via the formation of strong hydrogen bonds [67]. It was revealed that the confinement of AIEgens within the structure of the hyperbranched molecule restricted their intramolecular rotation and caused increased photon emission from the assembly (Figure 4b). We found that a set of strong hydrogen bonds formed between the AIEgen and hyperbranched polymer, creating a highly fluorescent (×30 times) assembly with excellent stability, demonstrating a negligible decrease in fluorescence intensity over almost 7 days, compared to the AIEgen alone. This was evidenced by the equilibrium dissociation constant being greater in the assembly (K_d_ = 1 × 10^−9^) than that measured in the AIEgen alone with K_d_ = 1 × 10^−5^.

**Figure 5:**
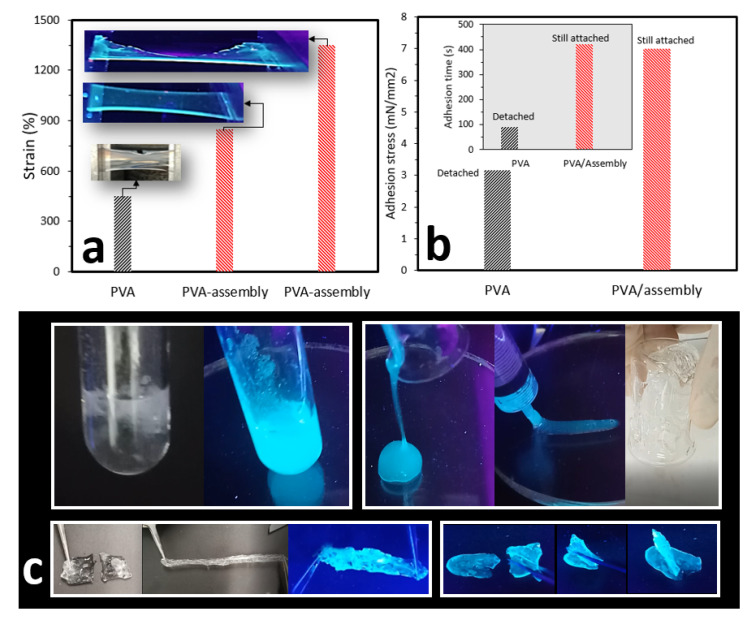
(**a**) Elongation at the break for PVA film compared to PVA containing AIEgen-hyperbranched assembly. (**b**) Upon crosslinking by 100 mM borax, PVA/hyperbranched assembly showed stronger self-healing properties with a greater adhesion time than that of the PVA alone. (**c**) Camera images of PVA gel, constructed using 50 mM borax, with and without exposure to a UV light at 350 nm (top left panel). When a VFD was employed, an injectable fluorescent PVA hydrogel was prepared, compared to the noninjectable PVA gel prepared traditionally (top right panel). A PVA film prepared by using 100 mM borax exhibited good self-healing properties with high extensibility (bottom panels). Images in (**c**) extracted from the supporting information (videos) from reference [67].

We further employed the assembly to create a fluorescent PVA hydrogel. By mixing the assembly with a PVA gel (6% *w/w*), using a solvent casting approach, a fluorescent PVA hydrogel film with excellent extensibility was obtained. Our results revealed that the strains at the breaking point for PVA and PVA containing AIEgen-hyperbranched assembly were 450% and 1350%, respectively, when under tensile displacement control testing (Figure 5a). Our previous study identified that regulating the concentration of borax in a VFD tube with rotation speed = 4000 RPM resulted in the creation of both PVA gels and films with self-healing properties with borax concentrations of 50 and 100 mM, respectively [66]. Following a similar approach, with borax concentrations = 50 and 100 mM, and VFD rotation speed = 4000 RPM, we introduced the AIEgen-hyperbranched assemblies into the PVA gel to successfully create injectable and self-healing fluorescent PVA hydrogel. Our results showed that PVA/assembly adhesive films, crosslinked by 100 mM borax, exhibited strong self-healing properties compared to the PVA alone, with more than 150% higher adhesion stress after 420 s tension testing. In contrast, PVA adhesive film was detached after 90 s (Figure 5b). PVA gel constructed from 50 mM borax was fluorescent under UV exposure. When a VFD was employed, an injectable fluorescent PVA hydrogel was prepared, compared to the noninjectable PVA gel prepared traditionally, indicating the crucial role of a VFD in the preparation of injectable fluorescent gels. A PVA film prepared using 100 mM borax exhibited good self-healing properties with high extensibility (Figure 5c).

The angle of the VFD tube was set to 45°, enabling us to compare different studies. In addition, different studies have shown that the tilt angle = 45° resulted in a more efficient outcome for material synthesis. For the creation of fluorescent hydrogels, we observed that higher rotation speeds resulted in the formation of smaller AIEgens, facilitating their penetration into the hyperbranched polymer structure and enhancing the final fluorescence property. Therefore, the rotation of the VFD tube was set to 4000 RPM in all experiments, and mechanical evaluations were performed on the hydrogels that were developed at that rotation.

## 3. Future Outlook

In this minireview, we introduced VFD as a new platform enabling one-step fabrication of hydrogels with different surface morphologies. We also described the application of VFD in the creation of fluorescent hydrogels with excellent photostability and fluorescence properties, and detailed tuning of the structure–property relationship in hydrogels. We believe that future studies, based on this minireview, will provide new opportunities to develop hydrogels with superior properties for different biomedical and engineering applications. Because our preliminary studies involved mainly physically crosslinked hydrogels, one direction for future studies is to investigate the impact of a VFD on the development of chemically crosslinked hydrogels and to develop an understanding of whether a VFD successfully tunes their surface morphologies and associated properties. A VFD provides an outstanding platform for the micromixing of chemicals under precisely controllable shear stress. Moreover, the VFD has proved to upscale under continuous flow conditions, and with the recent commissioning of a much larger VFD (50 mm OD versus 20 OD for earlier versions of the device). It is believed that future studies can take advantage of this opportunity to develop nanohybrid hydrogels with the ability to tune their properties during micromixing. Currently available technologies providing high shear rates during mixing, such as homogenizers, have utilized different flow dynamic routes compared to that of a VFD. Using a VFD shear stress is applied in Stewartson/Ekman thin layers with Faraday waves governing the fluid dynamics. Moreover, it is possible to apply different external fields such as electrical, magnetic, plasma, and laser to further enhance the process. This capability to apply different fields during micromixing is key to the creation of advanced hydrogels with new structural features and properties. Future studies can be directed towards the preparation of new fluorescent probes to create further fluorescent hydrogels. The combination of AIEgens with different materials to develop new fluorescent probes at the nanoscale has potential applications beyond hydrogels and will advance the field of bioimaging and sensing.

## 4. Conclusions

As a direct result of the current minireview, a VFD technology was introduced to create physically crosslinked hydrogels with superior fluorescence and self-adhesion properties and different morphologies. The precise micromixing of components, which was readily achievable with the use of the VFD, led to better spatial distribution, creating advanced hydrogels with more uniform structures compared to their traditionally prepared counterparts. It is believed that future studies, based on this minireview, will provide new opportunities to develop hydrogels with superior properties and different surface morphologies for different biomedical and engineering applications.
